# Molecular analysis of NPAS3 functional domains and variants

**DOI:** 10.1186/s12867-018-0117-4

**Published:** 2018-12-03

**Authors:** Leiah M. Luoma, Fred B. Berry

**Affiliations:** 1 Department of Medical Genetics, University of Alberta, Edmonton, AB Canada; 2 Department of Surgery, 3002D Li Ka Shing Centre, University of Alberta, Edmonton, AB T6G 2E1 Canada

**Keywords:** NPAS3, Transcription factor, bHLH–PAS, ARNT, VGF, TXNIP

## Abstract

**Background:**

*NPAS3* encodes a transcription factor which has been associated with multiple human psychiatric and neurodevelopmental disorders. In mice, deletion of *Npas3* was found to cause alterations in neurodevelopment, as well as a marked reduction in neurogenesis in the adult mouse hippocampus. This neurogenic deficit, alongside the reduction in cortical interneuron number, likely contributes to the behavioral and cognitive alterations observed in *Npas3* knockout mice. Although loss of Npas3 has been found to affect proliferation and apoptosis, the molecular function of NPAS3 is largely uncharacterized outside of predictions based on its high homology to bHLH–PAS transcription factors. Here we set out to characterize NPAS3 as a transcription factor, and to confirm whether NPAS3 acts as predicted for a Class 1 bHLH–PAS family member.

**Results:**

Through these studies we have experimentally demonstrated that NPAS3 behaves as a true transcription factor, capable of gene regulation through direct association with DNA. NPAS3 and ARNT are confirmed to directly interact in human cells through both bHLH and PAS dimerization domains. The C-terminus of NPAS3 was found to contain a functional transactivation domain. Further, the NPAS3::ARNT heterodimer was shown to directly regulate the expression of *VGF* and *TXNIP* through binding of their proximal promoters. Finally, we assessed the effects of three human variants of NPAS3 on gene regulatory function and do not observe significant deficits.

**Conclusions:**

NPAS3 is a true transcription factor capable of regulating expression of target genes through their promoters by directly cooperating with ARNT. The tested human variants of NPAS3 require further characterization to identify their effects on NPAS3 expression and function in the individuals that carry them. These data enhance our understanding of the molecular function of NPAS3 and the mechanism by which it contributes to normal and abnormal neurodevelopment and neural function.

**Electronic supplementary material:**

The online version of this article (10.1186/s12867-018-0117-4) contains supplementary material, which is available to authorized users.

## Background

*NPAS3* [Neuronal PAS (period-ARNT-single minded)-domain containing 3] encodes a transcription factor of the basic Helix Loop Helix–PAS (bHLH–PAS) family expressed in the developing central nervous system [1, 2]. *NPAS3* was originally characterized in humans as the causative locus of intellectual disability and psychosis in a Scottish family, as it was broken by a reciprocal translocation that segregated with disorder [1, 3]. Since its discovery as a potential “schizophrenia gene”, *NPAS3* has been robustly associated with neurodevelopmental and neuropsychiatric disorders commonly characterized by alterations in white matter connectivity, and intellectual disability. Large scale deletions including NPAS3 have been associated with holoprosencephaly, holoprosencephaly microform and other gross neurodevelopmental abnormalities [4–6]. Smaller deletions physically limited to *NPAS3* have been reported as associated with intellectual impairment and disorders of psychosis [7, 8]. Genome-wide studies have identified *NPAS3* as associated with bipolar disorder and schizophrenia [9–11]. Single nucleotide variation affecting the coding regions of *NPAS3* have been associated with neuropsychiatric disorders, including schizophrenia [12, 13]. These data are strongly suggestive of a role for NPAS3 in normal neurodevelopment and neuropsychological function.

The effect of loss of *Npas3* has been studied using mouse knockout models which have identified deficits in neurodevelopment resulting in altered neuroanatomy, as well as an almost complete loss of adult neurogenesis in the dentate gyrus of the hippocampus [14, 15]. Disruption *Npas3* expression was found to contribute to behavioral deficits which include hallmarks of hippocampal dysfunction, including reduced performance on tasks dependent on hippocampal memory, as well as altered emotional tone [14, 15]. Deletion of *Npas3* was found not to result in reduced proliferation of neuroprogenitors in the dentate gyrus of the hippocampus, but instead in increased markers of apoptosis [16]. During development, *Npas3* deletion results in reduced formation of cortical interneurons born in the subpallial ganglionic eminences [17]. As such, NPAS3 appears to be critical for neurogenic processes, with potentially far-reaching effects.

Since its discovery, NPAS3 has been characterized as a bHLH–PAS transcription factor based on predicted functional domains [2, 18]. bHLH–PAS proteins contain a bHLH DNA binding and protein interaction domain, followed by PAS domains, two degenerate repeat 70 aa domains, which are involved in protein interaction and ligand binding [19, 20]. A transactivation domain or repressive domain may be encoded C-terminal to the bHLH and PAS domains. All bHLH–PAS proteins are thought to act as heterodimers, requiring interaction with a general heterodimeric partner, such as ARNT, to create a functional heterodimer capable of regulation of target genes [19]. These heterodimers interact through residues in both the bHLH and PAS domains, where the PAS domains specify the interaction partner, while the bHLH domains are able to homo- and heterodimerize with other bHLH-containing proteins [21].

Our understanding of the molecular mechanism of gene regulatory function driven by NPAS3 is lacking. Aryl hydrocarbon receptor nuclear translocator (ARNT) has long been considered to be the obligate heterodimeric partner of NPAS3, however, the interaction between NPAS3 and ARNT had not been molecularly assessed until recent studies of mouse Npas3 and Arnt [22, 23]. NPAS3 and ARNT have been shown to cooperatively regulate genes involved in fibroblast growth factor (FGF) and sonic hedgehog (SHH) signaling, however whether this cooperation involved physical interaction was not demonstrated [24]. Microarray studies in human cells identified hundreds of target genes differentially regulated by expression of NPAS3, however, which are direct targets was not assessed [25]. Of the genes identified in this study, *VGF* (non-acronymic) has been shown to be regulated by NPAS3 in a manner dependent on constructs proximal to the promoter region [25, 26]. Neither physical association with the promoter region, nor the contribution of ARNT to this regulation were assessed. Recent ChIP-seq (chromatin immunoprecipitation next generation sequencing) studies have identified multiple targets of Npas3 which are differentially regulated in the mouse hippocampus, however the mechanism by which Npas3 regulates these genes is unknown [27]. In this study we performed experiments to characterize the functional domains of NPAS3 in protein interaction and gene-regulatory function. We assessed the relative contribution of the bHLH, PAS and C-terminal putative transactivation domains to these functions. Furthermore, we generated three variants previously identified in the human population, including two psychiatric illness-associated variants p.Val304Ile (c.910G>A, rs146677388) and p.Ala552Pro (c.1654G>C, rs12434716), and one rare population variant, p.Gly697Ser (c.2089G>A, rs141427321), for effects on NPAS3 function.

## Methods

### Plasmids

**Fig. 1:**
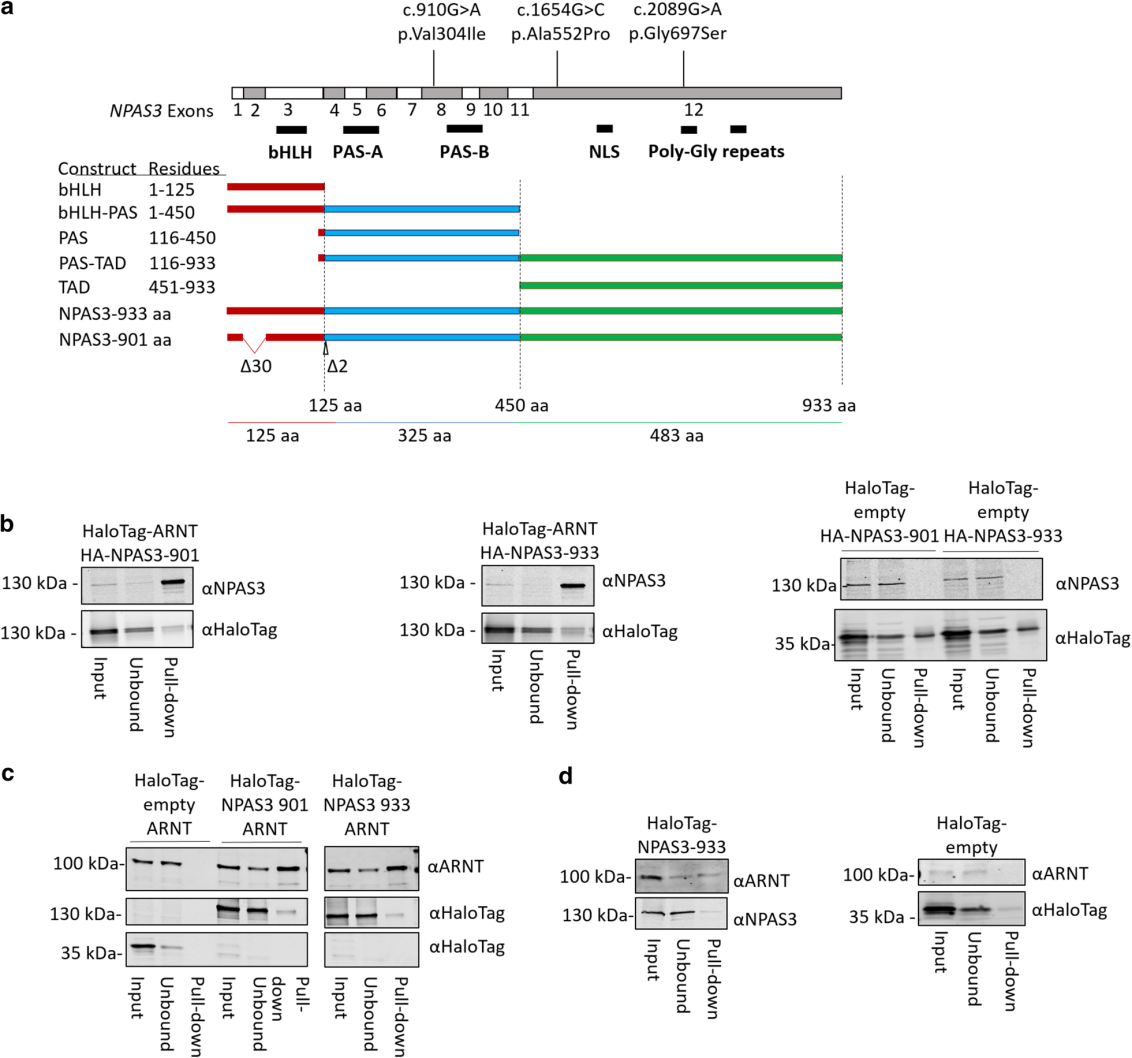
NPAS3 and ARNT interact through their bHLH and PAS domains. **a** Ideogram of NPAS3 constructs used. Exon numbering relative to *NPAS3* transcript variant 1 corresponding to NPAS3 isoform 1 (933 aa). Only coding regions of exons are depicted. Domain constructs and variant numbering are relative to isoform 1. NPAS3 isoform 2 (901 aa) was also characterized. *Red* bHLH domain, *Blue* PAS domains, *Green* TAD (C-terminal transactivation domain). **b** Western blots of HaloTag pull-downs demonstrating that all tested isoforms of NPAS3 and ARNT are able to robustly interact, which is not observed with the empty HaloTag vector. Bands in the top panel of pull-down lane of ‘prey’ protein, indicating physical association of HA-NPAS3 isoforms with the HaloTag-ARNT construct. Middle and bottom panels are results for the HaloTag-clone and HaloTag-empty constructs. As HaloTag constructs are covalently linked to the resin, they will be depleted in the pull-down sample, demonstrating functionality of the HaloTag. Molecular weight of HaloTag-ARNT, 121 kDa, HaloTag construct with no cDNA: 34 kDa, Full-length NPAS3: 101 kDa. **c** Reciprocal HaloTag pull down data using HaloTag-NPAS3 constructs and untagged ARNT isoform 1. Molecular weight of HaloTag-NPAS3, 135 kDa. **d** HaloTag-NPAS3 pull-down of endogenously expressed ARNT. All pull-downs have been performed at least twice

The coding sequence of *NPAS3* transcript variants 1 (NM_001164849.1 encoding isoform 1 NP_001158221.1, 933 aa) and 2 (NM_022123.1 encoding isoform 2 NP_071406.1, 901 aa) were purchased from Origene and cloned using the Gateway subcloning system (Invitrogen) into a Gateway converted pcI-HA (hemagglutinin tagged) vector. pHTN-CMV-neo (Promega) was also converted to the Gateway system and *NPAS3* was subcloned into it to generate a HaloTag construct. HaloTag-*ARNT* transcript variant 6 (NM_001286036.1 encoding isoform 6 NP_001272965.1 with a silent substitution of c.768G>C, p.Val188 = that is not predicted to change the protein) was purchased (Promega), and pcDNA3.1-ARNT transcript variant 1 (NM_001668.3 encoding isoform 1 NP_001659.1) was acquired from DNASU [28]. Domain constructs of NPAS3 were generated from transcript variant 1 to isolate the bHLH domain (residues 1–125), PAS domains (residues 116–450) and C-terminus (residues 451–933) (Fig. 1a). Domains were cloned into a Gateway converted pcI-HA to generate N-terminally HA-tagged constructs.

Variants tested were generated through site directed mutagenesis of the clone of *NPAS3* transcript variant 1 in the Gateway pDONR221 vector (Invitrogen). The c.1654G>C (p.Ala552Pro) and c.2089G>A (p.Gly697Ser) variants were generated using the KapaHiFi Hot Start kit (Kapa Biosystems) and the following primers: (*NPAS3*-G1654C-F 5′-CGGTGCTCTGGGCCCGATGCAGATCAA-3′, *NPAS3*-G1654C-R 5′-TTGATCTGCATCGGGCCCAGAGCACCG-3′, G2089A-F 5′-CCCGCAGGGCAGCGGCGGTGG-3′, *NPAS3*-G2089A-R 5′-CCACCGCCGCTGCCCTGCGGG-3′, variant nucleotides underlined). The c.910G>A (p.Val304Ile) variant was generated using a synthesized 504 bp gene block (IDT DNA) between the *Avr*II and *Xho*I (both NEB) sites in the *NPAS3* clone, containing the indicated variant. The variant was assembled into digested pDONR221-*NPAS3* transcript variant 1 using the Gibson Assembly Cloning Kit (NEB), and recombined into the pcI-HA destination vector.

### Cell culture and transfections

HEK 293T cells were purchased from ATCC and cultured in Dulbecco’s Modified Eagle Medium (DMEM) with high glucose (Sigma) in a 37 °C incubator with a humidified 5% CO_2_ atmosphere. Cells were subcultured at a ratio of 1:5 to 1:10 every 2–3 days, once they reached 75% confluence. For experiments, cells were plated at 2.2 × 10^6^ cells per 10 cm plate and allowed to recover for 24 h before transfection. Transfections were performed using Mirus TransIT LT-1 or Mirus TransIT Express (LT-1 replacement, MirusBio) per the manufacturer’s protocol. Briefly, 4 μg of total transfected DNA was used per reaction, with 12 μl of transfection reagent in 500 μl of serum-free media. Transfection reactions were mixed and incubated for 30 min at room temperature and added dropwise to cells. Cells were harvested 48 h after transfection.

### Immunoblot and protein::protein interaction studies

Transfected HEK 293T cells were washed twice and collected by centrifugation at 800×*g* in PBS (phosphate buffered saline) and frozen to enhance lysis. Cells were thawed, lysed with Mammalian Lysis Buffer (Promega) supplemented with protease inhibitor cocktail (Promega) and 1 mM PMSF (phenylmethylsulfonyl fluoride) per the manufacturer’s protocol. Samples were kept on ice during purification. Insoluble material was precipitated by centrifugation at 10,000×*g* for 5 min at 4 °C. For western blotting, samples were quantified using the BioRad Protein assay (BioRad) and spectrophotometry at 600 nm and 50 μg of protein was run per sample. Pull down reactions were performed using the HaloTag Mammalian Pull-Down protocol (Promega) per manufacturer’s instructions. Samples were reserved for analysis of the input, flow-through and pull-down samples.

Protein lysates and HaloTag pull-down samples were electrophoresed on 8–15% polyacrylamide gels at 140 V until the dye front reached the end of the gel. Proteins were transferred to nitrocellulose membranes using wet transfer with Towbin buffer. Transfers were run at 30 V overnight at 4 °C. Blots were rinsed with water and blocked for 1 h in LI-COR Block (LI-COR), before being probed with primary and secondary antibodies for 1 h each, with four washes in PBS-Tween buffer after each antibody. Blots were probed with the following antibodies: αHaloTag (mouse, Promega G921A) at 1/10 000, αNPAS3 (guinea pig, generated by Pocono Rabbit Farms & Laboratory to custom oligopeptide C-terminal to the PAS domain, validation shown in Additional file 1: Figure S1) at 1/10 000, αARNT (rabbit, Cell Signaling Technologies D28F3), donkey anti rabbit AlexaFluor 680 (Life Technologies A10043) at 1/25 000, goat anti-guinea pig IRDye 800 (Rockland 606 132 129) at 1/25 000, donkey anti mouse AlexaFluor 680 (Life Technologies A10038) at 1/25 000. Probed blots were scanned using the LI-COR Odyssey scanner using default scan parameters, and processed using LI-COR Image Studio software.

### Immunofluorescence

HEK 293T were plated in 6-well plates with sterile coverslips at 3 × 10^5^ cells per well and allowed to recover for 24 h before being transfected with 1 μg of DNA and 3 μl of Mirus TransIT LT1 (MirusBio). Forty-eight hours after transfection, cells were fixed in 2% paraformaldehyde buffered in PBS for 20 min, washed twice in PBS 0.05% Triton-X100 (PBSX) and blocked in PBSX 5% BSA for 15 min. Coverslips were probed with primary antibodies αHA (mouse, Santa Cruz sc-7392, 1/500) and αARNT (rabbit, Cell Signaling Technologies D28F3, 1/250) for 1 h. Coverslips were washed twice and probed with donkey anti-mouse AlexaFluor594 (Invitrogen A21203, 1/1000) and donkey anti-rabbit AlexaFluor488 (Invitrogen A21206, 1/1000) for 1 h. Coverslips were washed twice and stained with DAPI (4′,6-diamidino-2-phenylindole, 2 μg/ml) for 5 min before mounting onto slides with ProLong Antifade Gold reagent (Invitrogen). Slides were visualized using a Leica DM RE fluorescent microscope and images processed using Northern Elite Eclipse (EMPIX), and ImageJ [29] to add scale bars.

### Identification of potential co-targets of NPAS3 and ARNT

The genes identified as differentially regulated by NPAS3 in a previous microarray study were cross-referenced to the Encyclopedia of DNA Elements (ENCODE) ChIP-seq data for ARNT in K562 cells (experiment ID ENCSR155KHM) visualized in the UCSC genome browser [25, 30, 31]. Potential co-targets were selected for screening based on the presence of peaks in both replicates and pooled analysis of the ENCODE ChIP-seq study, as well as peak identification by conservative and optimal peak calling algorithms, resulting in a score out of 5. *VGF* was also selected for assessment due to deeper characterization in the index microarray study [25].

### Gene expression analysis

HEK 293T cells were harvested 48 h post-transfection using the RNeasy mini kit (QIAGEN) per the manufacturer’s protocol. RNA samples were quantified by Nanodrop and 500 ng was used as input into the Quantitect RT kit (QIAGEN) for cDNA synthesis per the protocol. qPCR was performed on 0.5 μl of the resultant cDNA per reaction using the KAPA SYBR FAST Universal 2X qPCR master mix (KapaBioscience) per the manufacturer protocol, with 1 μl of 2 μM primers per reaction. PCR reactions were performed in triplicate. Cycling was performed in a CFX96 Touch (BioRad) with parameters as follows: 3 min initial denaturation at 95 °C, followed by 40 cycles of 5 s denaturation at 95 °C with 25 s extension at 60 °C. All qPCR data were normalized to three housekeeping genes: hydroxymethylbilane synthase (*HMBS*), hypoxanthine phosphoribosyltransferase 1 (*HPRT1*) and succinate dehydrogenase complex flavoprotein subunit A (*SDHA*). Primers used are listed in Additional file 2: Table S1.

### HaloCHIP

HEK 293T cells were plated and transfected with HaloTag NPAS3 isoform 1 with and without ARNT isoform 1, or HaloTag-ARNT isoform 6 with and without NPAS3 isoform 1 as described above. Forty-eight hours after transfection, a representative plate was counted, and 1 × 10^7^ cells were input per reaction. Cells were fixed for 10 min in 1% formaldehyde and cross-linking was quenched with 0.125 M glycine for 5 min. Cells were washed twice with PBS and collected in 1 mL PBS. Lysis was performed per the HaloCHIP protocol with the optional cytoplasmic lysis step and homogenization using a Dounce homogenizer and 25 passes of the B-pestle. Chromatin was sheared by sonication and benzonase digestion based on the protocol outlined in [32], using four cycles of sonication with a Biodisrupter probe sonicator at medium high (60%) intensity for 30 s on/off. MgCl_2_ was added to a final concentration of 1 mM followed by incubation for 15 min with 250 U of benzonase per reaction to result in shearing to 1 kb or smaller in size. ethylenediaminetetraacetic acid (EDTA) pH 8.0 was added to 5 mM to terminate the reaction. An input sample of 1% was reserved, and HaloCHIP was performed per the manufacturer’s instructions with slight modification. Blocked lysates were obtained by incubation of nuclear lysates with the HaloCHIP blocking reagent, a dye which is catalyzed by the HaloTag enzyme, terminally inhibiting its catalytic function, resulting in tagged constructs being unable to covalently bind the resin. The resin was washed with 2 ml of each wash indicated in the HaloCHIP protocol, including the optional high salt wash buffer. The first three washes included 5 mM EDTA pH 8.0 to ensure the benzonase was inactive during washing. Samples were eluted overnight in kit elution buffer supplemented with 5 mM EDTA pH 8.0 at 65 °C. DNA was purified using QIAquick gel extraction kit (QIAGEN) per the manufacturer’s protocol.

Enrichment was assessed using endpoint PCR and gel electrophoresis. From each ChIP sample, 1 μl was added to a master mix of the GoTaq green reaction buffer with 10 μM primers. Primers used are listed in Additional file 2: Table S2. Cycling conditions were as follows: denaturation at 95 °C for 2 min, 30 cycles of 95 °C for 30 s, 60 °C for 15 s, 72 °C for 15 s; amplification was completed by a 5 min incubation at 72 °C. PCR reactions were run on a 1.5% agarose 1X TBE (Tris borate EDTA) gel, stained with ethidium bromide, and visualized on a UV transilluminator.

### Reporter gene assay

Promoter constructs were generated synthetically using sequences designed on the UCSC genome browser human genome build hg19 [33]. Regions were selected to include HaloCHIP positive regions in our studies, ENCODE ARNT ChIP-seq peaks in the same interval, as well as binding sites for ARNT homo- and heterodimeric complexes predicted by ConTra v3.0 [34]. Binding sites for Hypoxia Inducible Factor 1 Alpha (HIF1A)::ARNT (MA0259.1), ARNT (MA0004.1) and Aryl Hydrocarbon Receptor (AhR)::ARNT (MA0006.1) were predicted in the 1000 bp proximal promoter, 5′UTR and intron 1 of *VGF* and *TXNIP* (thioredoxin interacting protein) using position-weight matrices from the JASPAR database indicated above, using the ConTra v3.0 settings: stringency core = 0.95, and similarity matrix = 0.85. For *VGF*, the promoter construct consisted of 797 bp upstream of the transcription start site as defined by NM_003378.3, as well as 13 bp of the first exon, as indexed in UCSC genome browser human genome build hg19. This region was synthesized (IDT DNA) for assembly into pGL4.10 linearized using *Xho*I and *Hin*dIII (both NEB) with the Gibson Assembly kit (NEB). For *TXNIP*, the region starting 867 bp upstream of the transcription start site defined by NM_006472.5, as well as 68 bp of exon 1, was cloned as described for *VGF*.

For luciferase, HEK 293T cells were plated at 5 × 10^4^ cells per well of a 24-well plate. Cells were plated and transfected in triplicate per condition. Cells were transfected using Mirus TransIT LT1 or TransIT Express (MirusBio) per the manufacturer’s protocol and 50 ng of pGL4.10- promoter-firefly luciferase, 150 ng of each driver construct, 0.1 ng pGL4.7-TK-renilla luciferase. Drivers were pcI-HA-NPAS3 isoform 1 and pcDNA3.1-ARNT isoform 1 or empty vectors (pcI-HA and pcDNA4). Forty-eight hours after transfection, cells were processed per the Dual-Luciferase Reporter Assay System (Promega) protocol. Luminescence was read using the GloMax Multi Jr Tube Multimode Reader (Promega) with the Luminescence module using the DLR-0INJ protocol. Reporter firefly luciferase luminescence was normalized to renilla luciferase luminescence to generate relative luminescence units (RLUs), calculated by the luminometer, to control for transfection efficiency.

### Statistics

For qPCR, quantification and statistical analyses were performed in the BioRad CFX manager 3.0 software. Graphs were generated in Excel 2016 (Microsoft). Sample variance was assessed in Excel 2016 (Microsoft) using Levene’s test and two sample t-tests were performed for pairwise comparisons. Differences in frequencies for immunofluorescence were calculated using χ^2^ tests.

## Results

### NPAS3 behaves as predicted for a Class 1 bHLH–PAS transcription factor

As NPAS3 is predicted to be bHLH–PAS transcription factor based on sequence conservation, notably to its orthologue *trachealess* and its closest paralogue Neuronal PAS-domain containing 1 (*Npas1*) [2], we set out to determine whether NPAS3 behaves as predicted for a Class 1 bHLH–PAS transcription factor. Class 1 bHLH–PAS proteins obligately interact with Class 2 bHLH–PAS proteins, such as ARNT, through their bHLH and PAS domains [19]. We assessed two isoforms of each NPAS3 and ARNT using the HaloTag system in HEK 293T cells to determine whether they interact with high affinity. Both isoforms of NPAS3 and ARNT were found to interact with one another (Fig. 1b, c). Isoform 1 and 2 of NPAS3, which differ by 30 aa and 2 aa flanking the bHLH DNA binding domain, were both found to interact with ARNT (Fig. 1a, b). Similarly, isoforms 1 and 6 of ARNT, which vary by two residues C-terminal to the bHLH and PAS domains, were also found to be able to interact with both NPAS3 isoforms (Fig. 1b, c). Further, expressed HaloTag-NPAS3 was able to pull-down endogenous ARNT, demonstrating specificity of this interaction (Fig. 1b, d). These data confirm that human NPAS3 and ARNT can interact, as observed for mouse Npas3 and Arnt [22, 23].

**Fig. 2:**
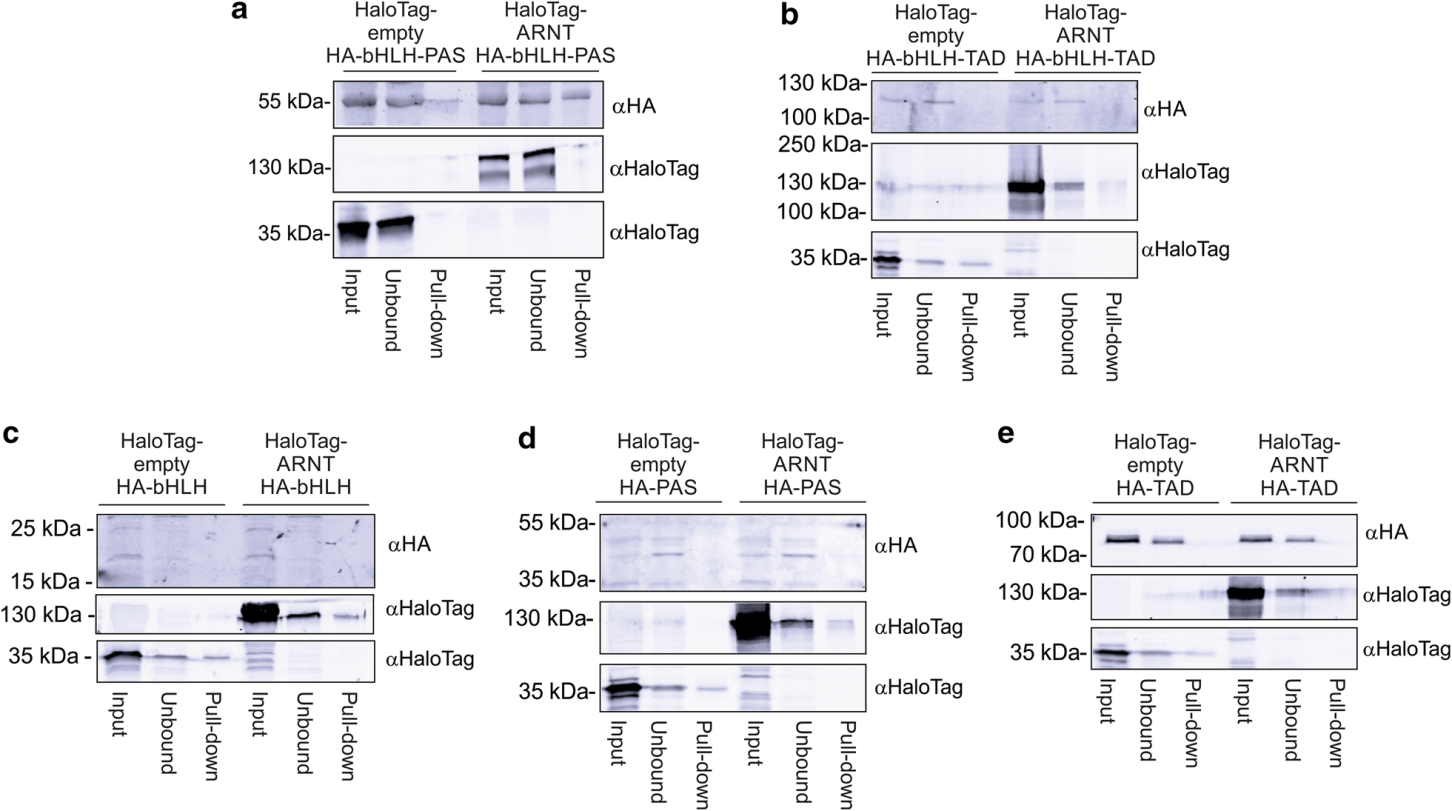
The bHLH and PAS domains are required for high-affinity interaction of NPAS3 and ARNT. Domain constructs used indicated in Fig. 1a. Western blots of HaloTag pull-down of HA-tagged NPAS3 domains by HaloTag-ARNT. Blots include lanes for input sample, unbound sample taken after binding reaction, and protein eluted off of resin after washing. Bands in the top panel of pull-down lane indicate physical association of HA-tagged NPAS3 domain construct with the HaloTag-ARNT construct. Middle and bottom panels are results for the HaloTag-ARNT and HaloTag-empty constructs. As HaloTag constructs are covalently linked to the resin, they will be depleted in the pull-down sample, demonstrating functionality of the HaloTag. Molecular weight of HaloTag-ARNT, 121 kDa, HaloTag construct with no cDNA: 34 kDa. Blot legend with expected sizes: **a** bHLH–PAS, 49.4 kDa, **b** PAS-TAD, 98.2 kDa, **c** bHLH, 13 kDa, **d** PAS, 35.8 kDa, **e** TAD, 53.2 kDa. TAD = C-terminal transactivation domain. Pull downs have been replicated at least twice

**Fig. 3:**
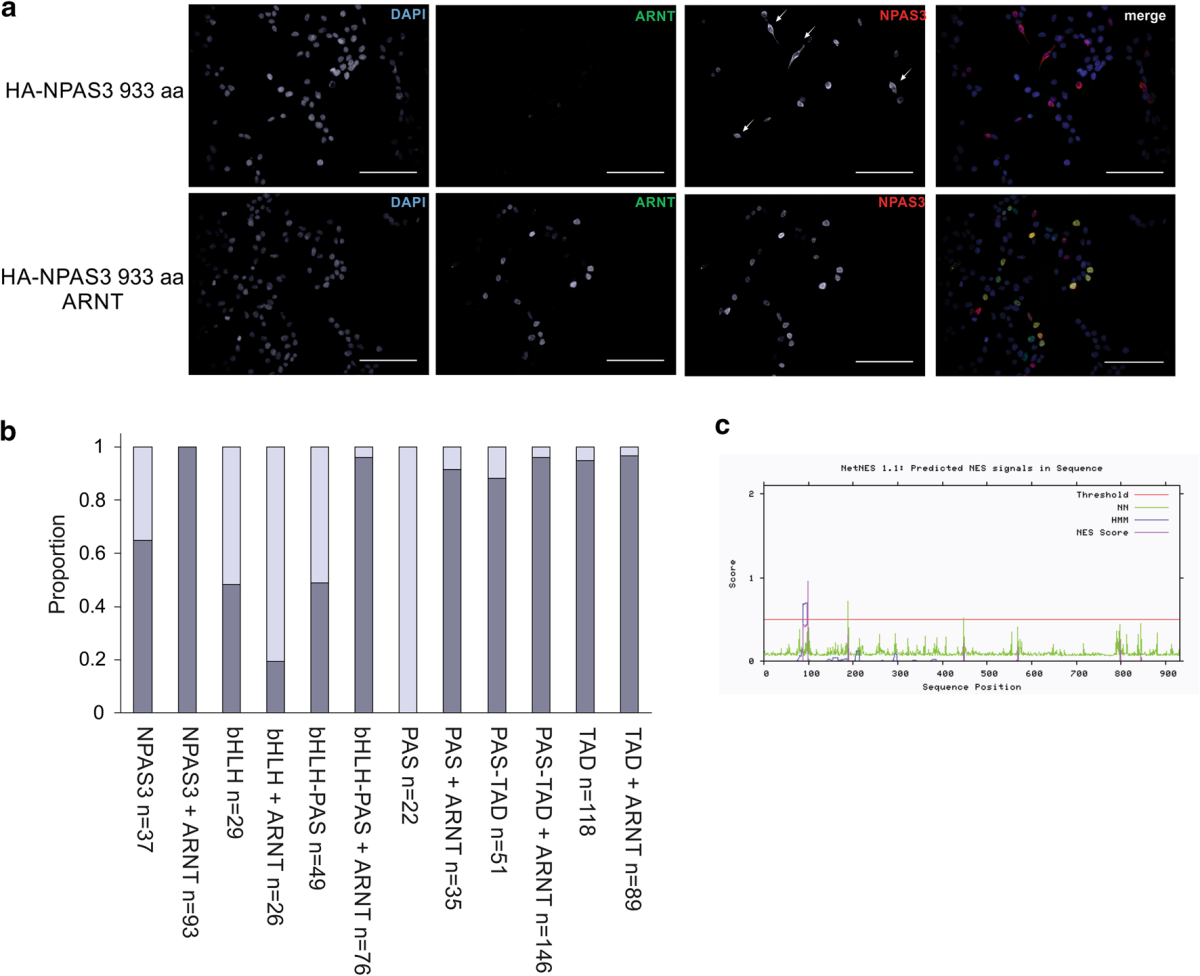
NPAS3 localization is affected by the region encoding the NLS and independently by ARNT. **a** Immunofluorescence images demonstrating that NPAS3 is predominantly localized to the nucleus, which is enhanced by co-expression of ARNT. Arrows indicate cytoplasmic localization of NPAS3. ×400 magnification, scale bar = 10 μm. **b** Quantification of localization of NPAS3 signal. Localization was scored as either nuclear or nuclear = cytoplasmic by an individual blinded to NPAS3 construct identity and to the presence or absence of ARNT. *p < 0.05, **p < 0.01, ***p < 0.001. **c** Output of NetNES 1.0 prediction software indicating a predicted NES in the region encoding the bHLH domain of NPAS3, indicated by scores above the threshold (red line)

In order to experimentally characterize the predicted functional domains of NPAS3, constructs expressing combinations of the bHLH, PAS and/or C-terminal putative transactivation domain were cloned from the NPAS3 isoform 1 coding sequence (Fig. 1a). The interaction with ARNT was found to require both the bHLH and PAS domains, but not the C-terminus (Fig. 2). Immunofluorescence was performed to determine whether domains are localized such that they are able to interact with ARNT. Full length NPAS3 was found to be cytoplasmic and nuclear localized, which was affected by co-expression of ARNT, where nuclear localization was enhanced with ARNT expression (Fig. 3, Additional file 3: Figure S2). All NPAS3 domain constructs were observed in the nucleus, with variable cytoplasmic localization (Fig. 3b). Constructs containing the C-terminus, where a nuclear localization sequence (NLS) has been predicted [13], were found to be predominantly localized to the nucleus in all conditions. Constructs containing the PAS domain were found to have enhanced nuclear localization with co-expression of ARNT. The bHLH domain demonstrated the opposite effect, which may be driven by a nuclear export sequence (NES) predicted in the bHLH domain by NetNES (Fig. 3b, c) [34]. These data suggest that NPAS3 localization may be actively regulated at multiple levels, which can affect gene regulatory output. In summary, our data experimentally validate the function of the predicted domains of NPAS3, and suggest that the observed variable localization of NPAS3 may be contributed to active regulation of NPAS3 localization.

### NPAS3 and ARNT co-regulate *TXNIP* and *VGF*

**Fig. 4:**
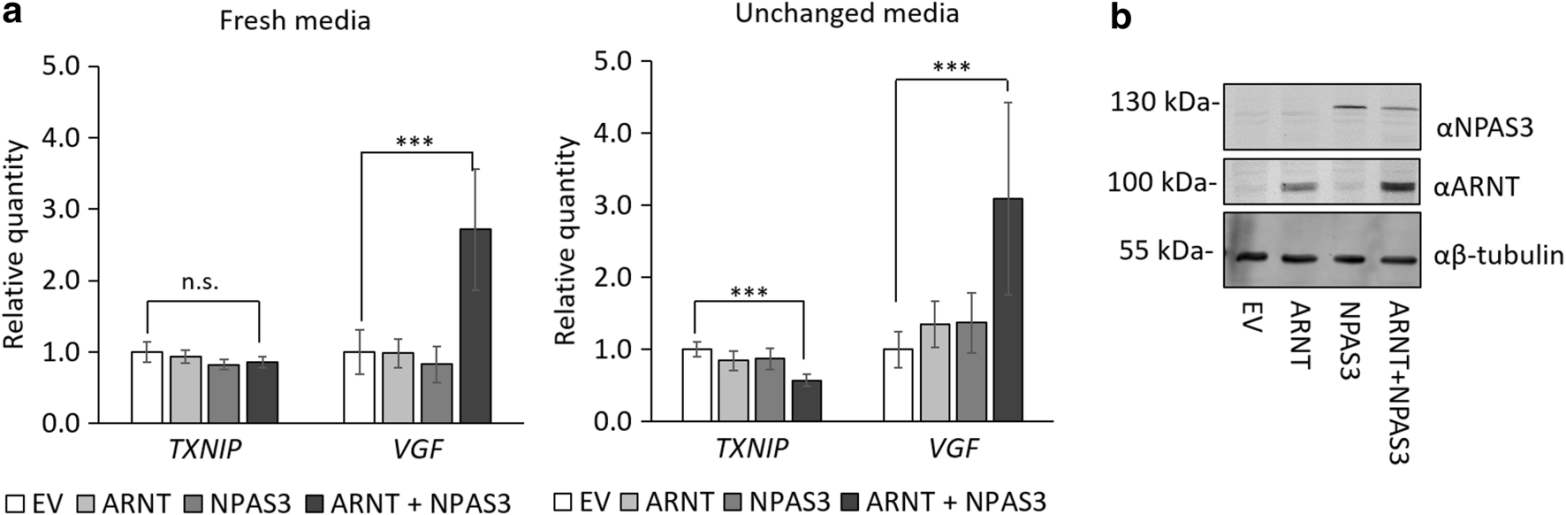
NPAS3 and ARNT regulate expression of *TXNIP* and *VGF.* **a** Relative quantity of mRNA as assessed by qRT-PCR in HEK 293T cells expressing NPAS3 or ARNT. Expression was assessed 48 h post-transfection, with media replacement 24 h post-transfection and without. *VGF* and *TXNIP* were found to be regulated in response to expression of NPAS3 and ARNT. *p < 0.05, **p < 0.01, ***p < 0.001. Data are representative of three biological replicates. **b** Western blot demonstrating expression of constructs indicated

As we were able to confirm the interaction between human NPAS3 and ARNT, we screened regulatory targets of NPAS3 for regions likely bound by ARNT to identify potential regulatory targets of the NPAS3::ARNT heterodimer. We screened all genes previously identified as differentially expressed in response to ectopic expression of NPAS3 in human cells for ARNT ENCODE ChIP-seq positive regions within 1 kb of the transcription start site [25, 28]. Thirteen genes were identified in our screen for potential co-targets of NPAS3 and ARNT, listed in Table 1. VGF was added as a potential target due to further characterization in other studies [25, 26]. Of these genes, two were found to be regulated by co-expression of NPAS3 and ARNT: *VGF* and *TXNIP* (Fig. 4). The direction of regulation of both *VGF* (up-regulated) and *TXNIP* (down-regulated) were found to be consistent with those observed in the index study identifying the regulatory effect [25], however, we did not observe regulation of these targets with singly expressed NPAS3, which was performed in the index study. *TXNIP* was found to be differentially regulated only in the relatively ‘stressed’ condition where media was not replaced 24 h post transfection, suggesting that this regulation may be stimulus specific. *VGF* was found to be up-regulated by co-expression of NPAS3 and ARNT in all conditions tested.

**Table 1:** Potential co-targets of NPAS3 and ARNT screened in this study

Gene name	ENCODE ARNT ChIP-seq peaks^a^	Fold regulation by NPAS3^b^	Other identifiers	Functions
*VGF*	3	2.92		Dendritic arborization, proneurogenic [35, 36]
*HIST1H4H*	5	2.05	Histone H4	G1-S transition, replication licencing factors [37]
*ATF5*	5	1.83		Cell cycle progression G1-S, stress response, cAMP signalling, SVZ neurogenesis [38, 39]
*DHCR24*	5	1.75	Seladin-1	Neuroprotective, oxidative stress, inflammation [40]
*ZBTB40*	5	1.74		Unknown
*NCLN*	5	1.70	Nicalin	Nicalin-NOMO complex, nodal signalling [41]
*MAT2A*	5	1.67		S-adenosylmethionine synthesis, hypoxia [42]
*USP49*	5	− 2.10		Deubiquitinase [43]
*ANG*	5	− 2.16	RNASE5	Alternate ORF transcribed from *RNASE4* [44]
*ZNF581*	5	− 2.24		Unknown
*RPL37*	5	− 2.33		p53 pathway, MDM2, MDMX [45]
*RNASE4*	5	− 2.40		Neuroprotective during oxidative stress [44]
*TXNIP*	5	− 2.57		Oxidative stress response, inflammation [46]
*ANKRD37*	5	− 4.49		Hypoxia [47, 48]

^a^Score out of 5 based on calling of peaks in all replicate analyses of the ARNT ChIP-seq data from the ENCODE project experiment ENCSR155KHM

^b^Regulation observed in [25]

### NPAS3 and ARNT physically associate with and regulate *VGF* through its promoter

**Fig. 5:**
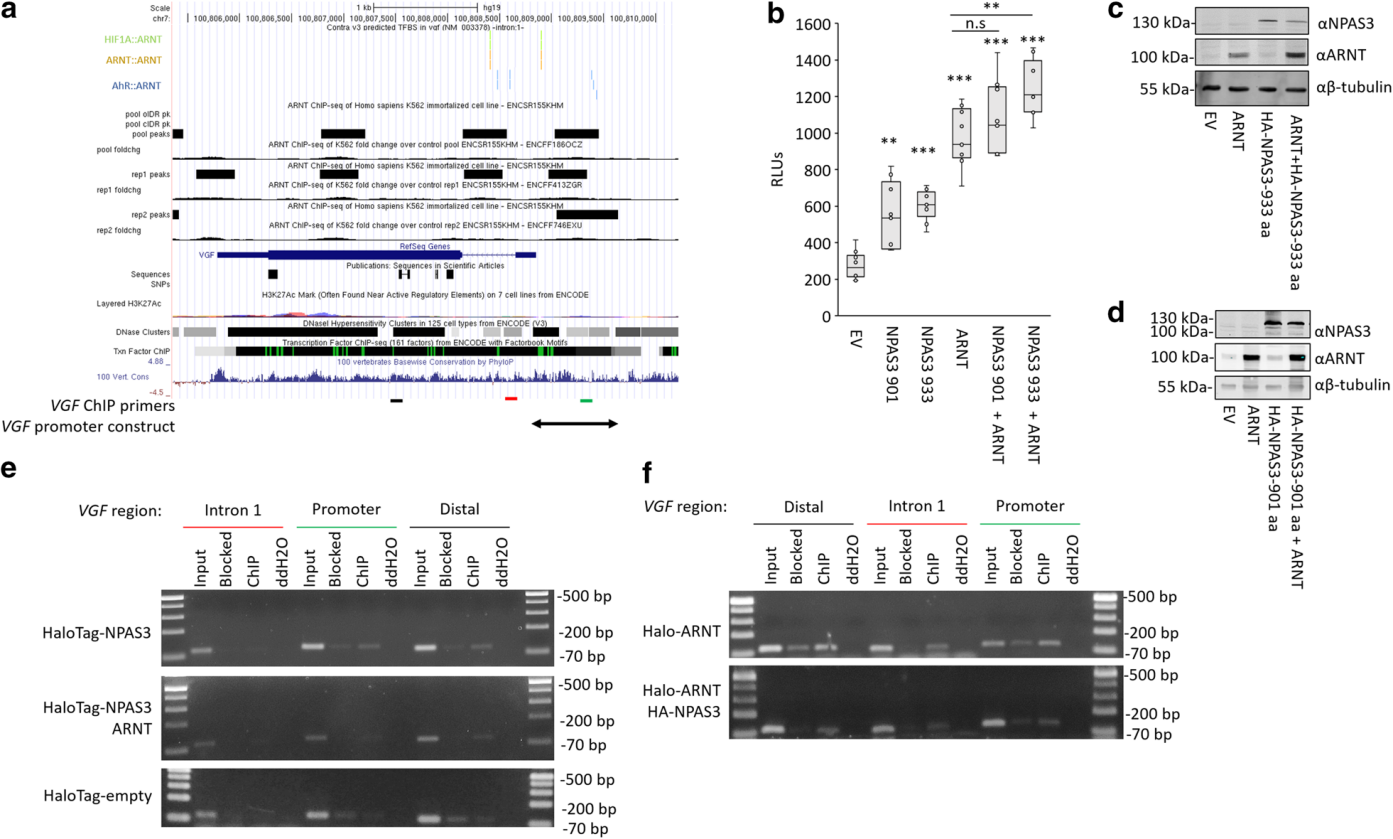
NPAS3 physically regulates the *VGF* promoter. **a** UCSC genome browser data used to identify potential NPAS3::ARNT binding sites proximal to the *VGF* locus. Accessed from http://genome.ucsc.edu. Top: ConTra v3.0 data indicating the location of predicted ARNT binding sites based on position weight matrices accessed from the JASPAR database. Middle: ENCODE ARNT ChIP-seq data indicating peaks proximal to the *VGF* locus. Bottom: relative localization of ChIP primers and promoter constructs used to assess regulation of this locus by NPAS3 and ARNT. VGF primers indicate regions amplified corresponding to Distal element (black line), Intron 1 (red line) and Promoter region (green line). **b** Luciferase data demonstrating that NPAS3 and ARNT regulate expression of the luciferase (*luc2*) reporter driven by a construct including 797 bp proximal to the transcription start site of *VGF*. RLU = relative luciferase units, the ratio of firefly luciferase and renilla luciferase to normalize for transfection efficiency. Top and bottom of each box indicate the 25th and 75th centile, internal bar indicates the median, whiskers indicate data extremes. Data are representative of three biological replicates. *p < 0.05, **p < 0.01, ***p < 0.001. **c**, **d** Western blots demonstrating expression of NPAS3 and ARNT constructs. **e** HaloCHIP PCR data demonstrating that NPAS3 interacts with the promoter region of *VGF* relative to other regions of the locus assayed. **f** HaloCHIP PCR data demonstrating ARNT signal at all regions tested at the *VGF* locus. HaloCHIP results are representative of three replicates

In order to determine if the observed regulation of *VGF* is due to association of the NPAS3 with the promoter, HaloCHIP, a variant of ChIP, was performed using HaloTag constructs of NPAS3 and ARNT. NPAS3 was found to bind regions proximal to the promoter of *VGF* (Fig. 5a, e). A second signal was observed in the distal region probed, which may represent an independent binding event as some signal is observed in the ENCODE ARNT ChIP-seq data (Fig. 5e). ARNT was found to associate with all regions probed at the *VGF* locus (Fig. 5f). To determine whether the observed binding to the interval upstream of the transcription start site results in the observed upregulation of *VGF* expression, transactivation assays were performed. Luciferase reporter expression was driven by a construct of the proximal promoter region of *VGF*. NPAS3 and ARNT were both found to activate reporter expression independently, with additive effects observed with co-expression of isoforms 1 of ARNT and NPAS3 (Fig. 5b). These data indicate that NPAS3 and ARNT physically associate with the *VGF* proximal promoter resulting in activation of expression.

**Fig. 6:**
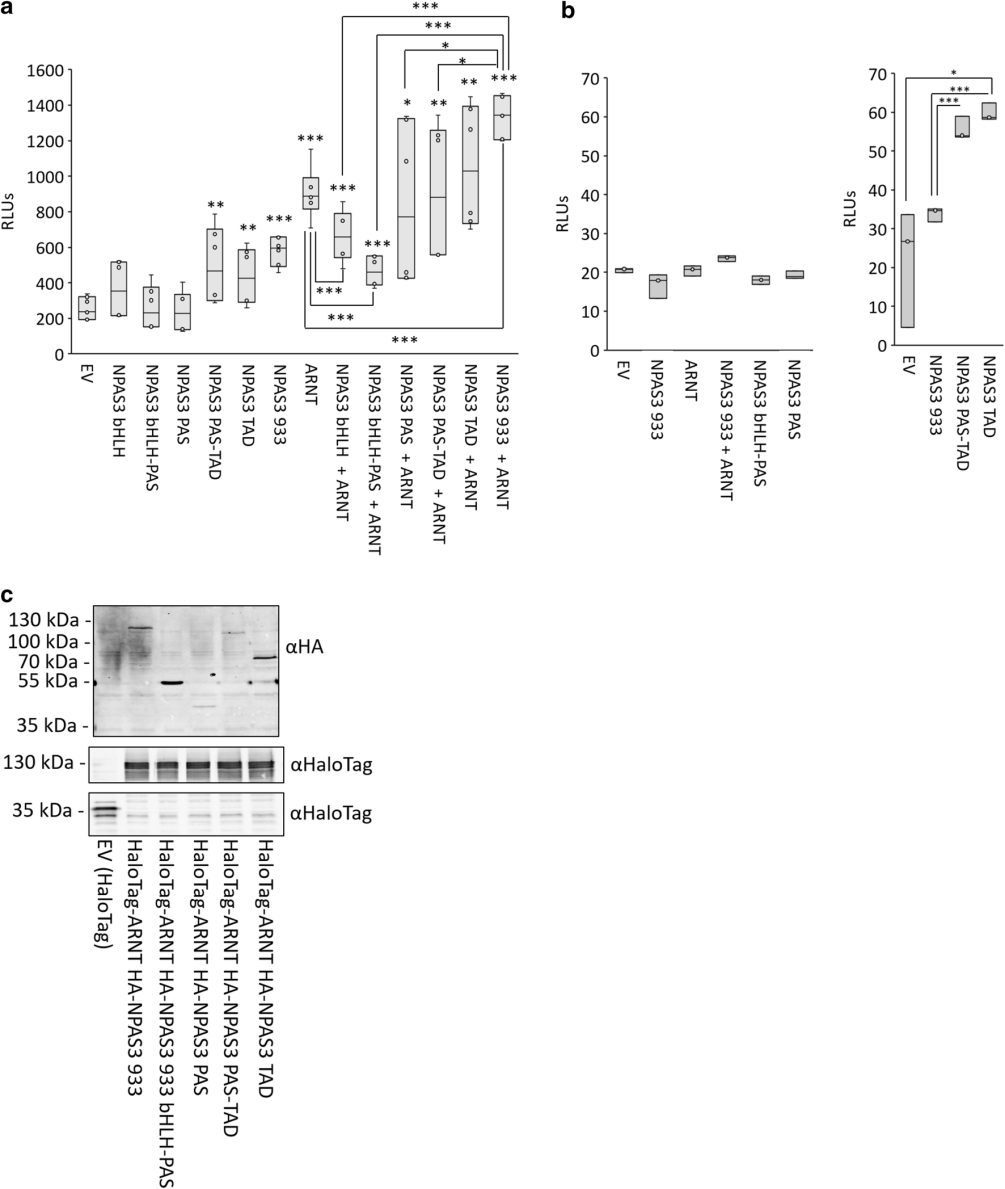
The contribution of NPAS3 domains to gene regulatory function. **a**, **b** Luciferase reporter assay demonstrating the gene-regulatory effects of NPAS3 domains. The C-terminus of NPAS3 is required to transactivate expression of a reporter construct, and without the bHLH and PAS domains the C-terminus drives non-specific transactivation. Reporter used is luciferase (*luc2*) expression driven by a construct including 797 bp proximal to the transcription start site of *VGF*. RLU = relative luciferase units, the ratio of firefly luciferase and renilla luciferase to normalize for transfection efficiency. Top and bottom of each box indicate the 25th and 75th centile, internal bar indicates the median, whiskers indicate data extremes. Data are representative of three biological replicates. *p < 0.05, **p < 0.01, ***p < 0.001. **c** Expression of NPAS3 domain constructs. Expected sizes: full length: 101 kDa, bHLH–PAS 49.4 kDa, PAS 35.8 kDa, PAS-TAD 98.2 kDa, TAD 53.2 kDa

As the *VGF* promoter construct is regulated by NPAS3 and ARNT, we used it to assess the gene-regulatory activity of the domains of NPAS3 (Fig. 6a). Singly-expressed NPAS3 constructs were not able to activate expression from this locus when the C-terminal predicted transactivation domain was deleted. When co-expressed with ARNT, the NPAS3 bHLH–PAS construct repressed the ability of ARNT to enhance expression of this reporter, supporting the ability of the bHLH–PAS domains to functionally interact with ARNT, and the C-terminus as required for transactivation function. The bHLH domain in isolation also appeared able to repress activation of this reporter by ARNT, potentially suggestive of some interactivity, or ability to compete with ARNT for binding to DNA targets. Assessment of transactivation from a control promoterless luciferase “empty vector” construct demonstrated that the activation observed by the full-length NPAS3 isoform 1 is specific to the *VGF* promoter region, and that the observed activation in the PAS-TAD and TAD (C-terminus, transactivation domain) constructs is likely due to potent but non-specific transactivation function (Fig. 6b). These data support the role of the C-terminus as a true transactivation domain, as well as of the bHLH domain for conferring sequence specificity of NPAS3 regulatory function.

### NPAS3 and ARNT physically associate with and regulate *TXNIP*

**Fig. 7:**
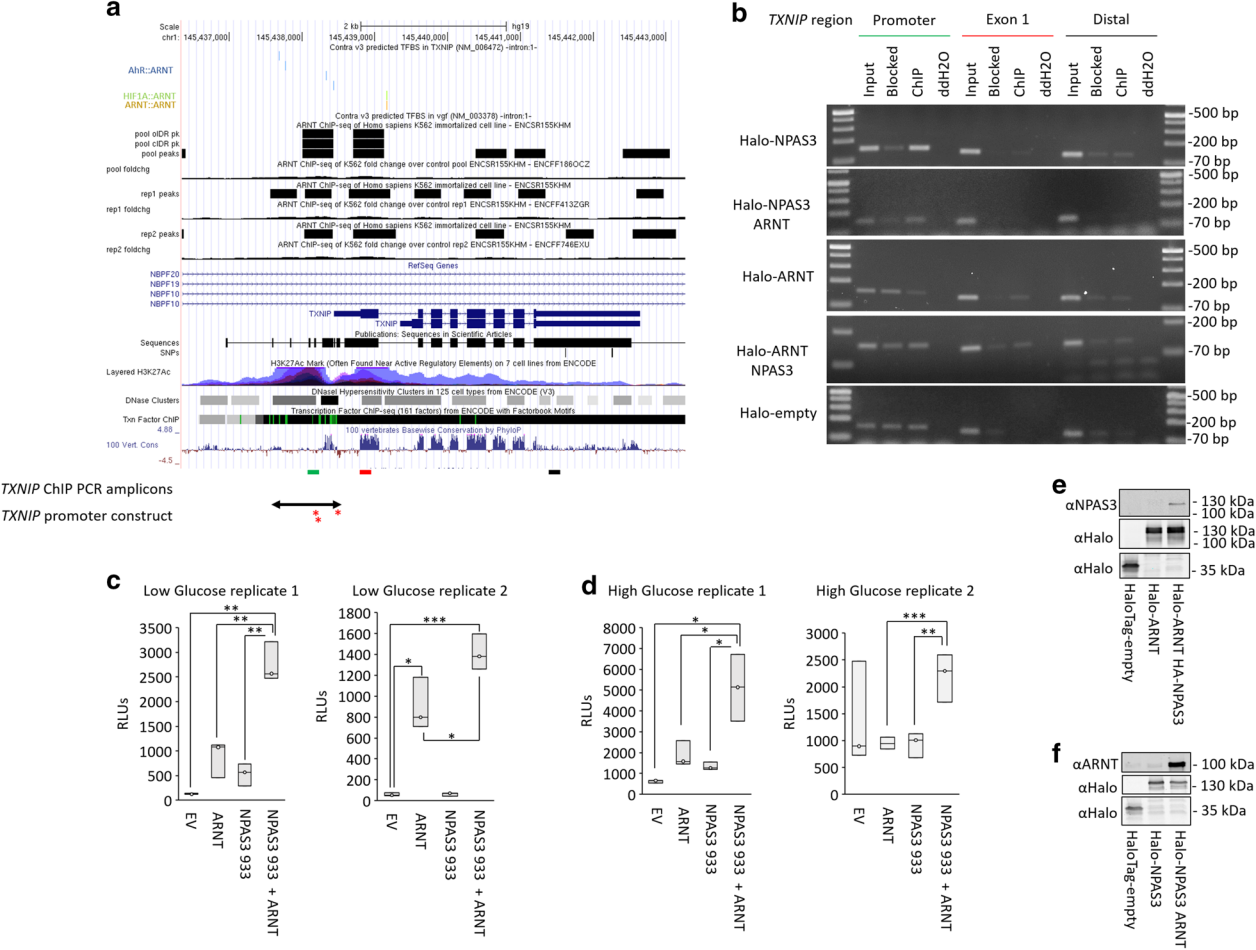
NPAS3 and ARNT physically regulate the *TXNIP* locus. **a** UCSC genome browser data used to identify potential NPAS3::ARNT binding sites proximal to the *TXNIP* locus. Accessed from http://genome.ucsc.edu. Top: ConTra v3.0 predicted ARNT binding sites in the promoter, 3′UTR and intron 1 of *TXNIP* based on position weight matrices accessed from the JASPAR database. Middle: ENCODE ARNT ChIP-seq data indicating peaks proximal to the *TXNIP* locus. Bottom: relative localization of ChIP primers and promoter constructs used to assess regulation of this locus by NPAS3 and ARNT. *TXNIP* primers indicate regions amplified corresponding to Distal element (black line), Exon 1 (red line) and Promoter region (green line). Red asterisks indicate variants in promoter construct relative to wild-type sequence. **b** Luciferase data demonstrating that NPAS3 and ARNT regulate expression of reporter luciferase (*luc2*) driven by a construct including 867 bp proximal to the transcription start site of *TXNIP*. RLU = relative luciferase units, the ratio of firefly luciferase and renilla luciferase to normalize for transfection efficiency. Top and bottom of each box indicate the 25th and 75th centile, internal bar indicates the median. Data are presented for two biological replicates performed in each low- and high-glucose conditions. *p < 0.05, **p < 0.01, ***p < 0.001. **c**, **d** Western blots demonstrating expression of NPAS3 and ARNT constructs. **e** HaloCHIP PCR data demonstrating that NPAS3 interacts with the promoter region of *VGF* relative to other regions of the locus assayed. **f** HaloCHIP PCR data demonstrating ARNT signal at all regions tested at the *VGF* locus. HaloCHIP results are representative of three replicates performed

We probed the NPAS3 and ARNT HaloCHIP samples to determine if the observed regulation of *TXNIP* is associated with physical association of NPAS3 and ARNT. NPAS3 was found to bind regions proximal to the promoter of *TXNIP* (Fig. 7a, b). Co-expression of ARNT resulted in a secondary signal observed in exon 1, and ARNT was not found to bind specifically associated with the promoter region without expression of NPAS3 (Fig. 7b). Luciferase reporter expression was driven by a *TXNIP* promoter construct in order to confirm that the observed regulation is driven by binding of NPAS3 this region. The *TXNIP* promoter could not be generated without secondary variants due to a repetitive C-rich region in the construct. These variants, relative to the transcription start site (Chr1:145,438,461) include − 246hetA/C (Chr1:145,438,215hetA/C), − 222ΔC (Chr1:145,438,239ΔC) and + 49T>C (Chr1:145,438,510T>C) and are indicated in Fig. 7a. These variants were found to lie at regions of low conservation, not predicted to affect ARNT binding, nor within regions characterized as involved in regulation of *TXNIP* in response to various environmental stimuli such as glucose and oxidative stress [35–39]. As such experiments were performed in low- and high-glucose conditions to confirm responsiveness of the construct to glucose. Co-expression of NPAS3 and ARNT were found to activate expression driven by this construct (Fig. 7c, d). The promoter was found to be responsive to glucose concentration, with higher baseline expression in high glucose relative to low glucose. The up-regulation driven by NPAS3 and ARNT was observed in both low- and high-glucose conditions. These data are opposite to the repression of the endogenous *TXNIP* locus by NPAS3 and ARNT co-expression observed by us, and in the index study identifying *TXNIP* as down-regulated by NPAS3 (Fig. 4, [25]). However, these data support physical association of NPAS3 and ARNT to the region encoded in this construct, despite the region cloned not being sufficient to assemble the repressive complex.

### Functional assessment of NPAS3 variants

**Fig. 8:**
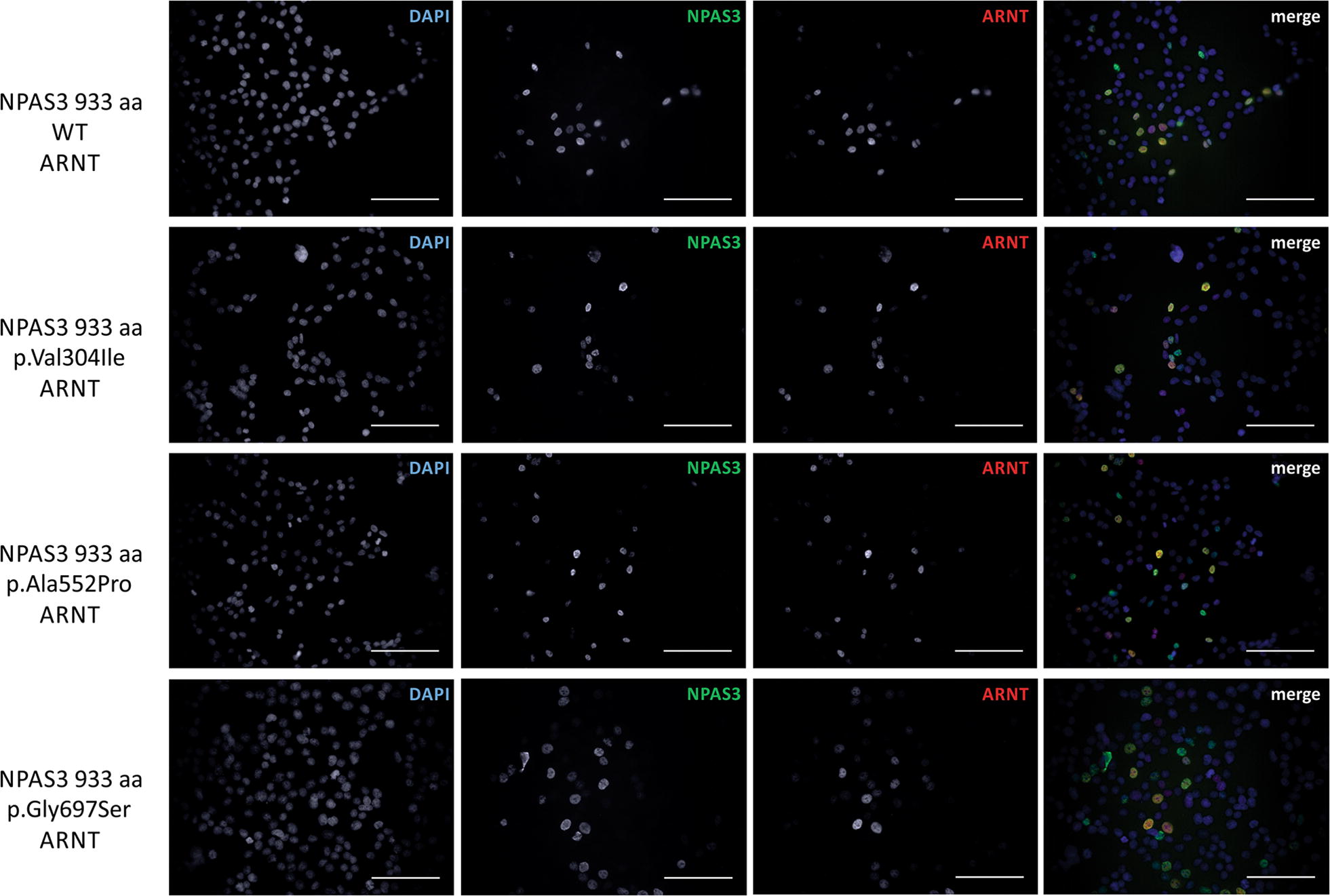
Localization of NPAS3 variants. Immunofluorescence microscopy images demonstrating that all NPAS3 variants are able to localize to the nucleus. HEK 293T cells were transfected with indicated constructs and incubated for 48 h prior to fixing with paraformaldehyde and probing for ARNT and NPAS3, and stained with DAPI to indicate nuclei. ×400 magnification, scale bar = 10 μm

**Fig. 9:**
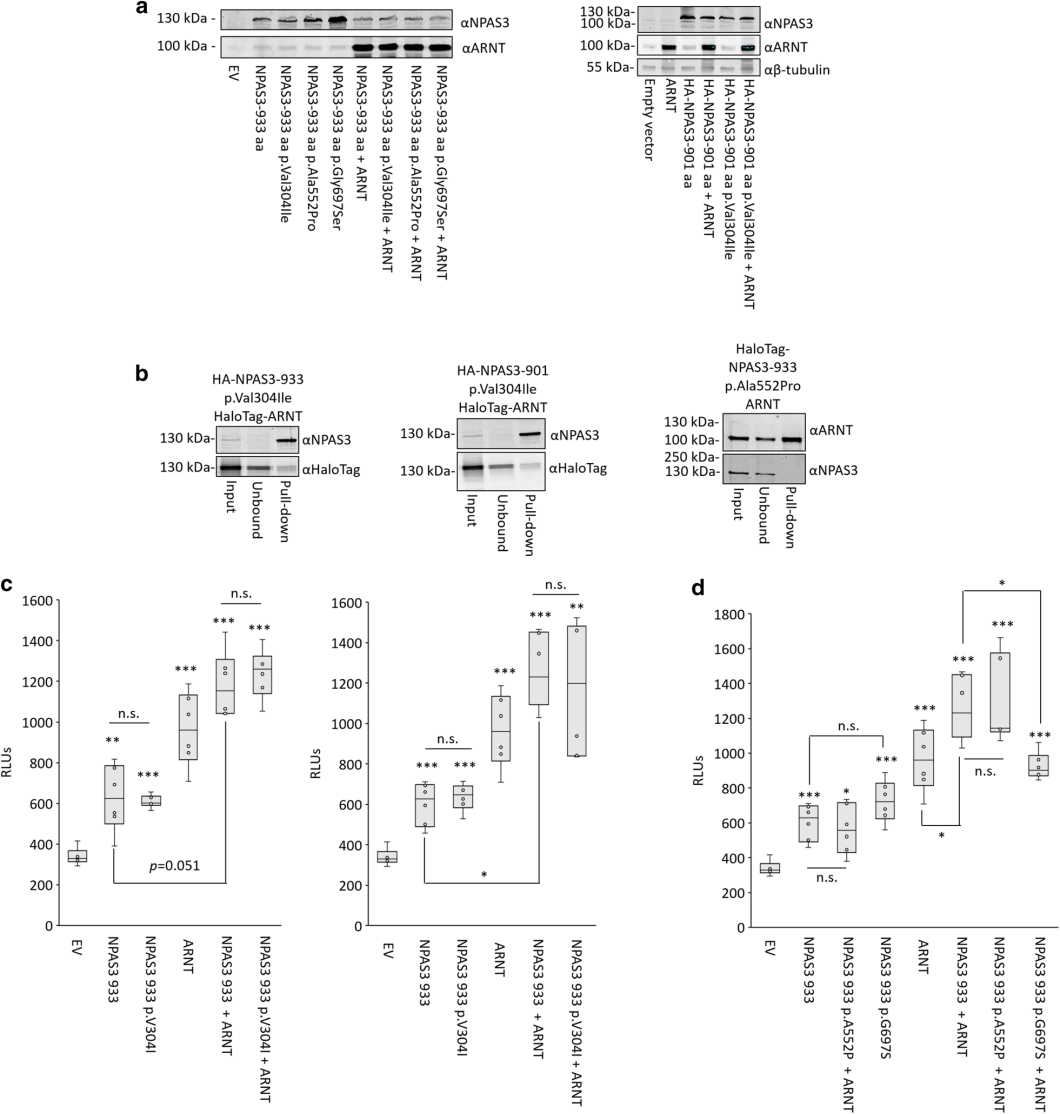
Functional characterization of NPAS3 variants. **a** Western blots showing that NPAS3 variants are normally expressed when transfected into HEK 293T cells. **b** HaloTag pull-down data demonstrating that the p.Val304Ile and p.Ala552Pro variants are able to interact with ARNT. Bands in the top panel of pull-down lane of ‘prey’ protein, indicating physical association of HA-NPAS3 variants with the HaloTag-ARNT construct. Middle and bottom panels are results for the HaloTag-clone and HaloTag-empty constructs. As HaloTag constructs are covalently linked to the resin, they will be depleted in the pull-down sample, demonstrating functionality of the HaloTag. Molecular weight of HaloTag-ARNT, 121 kDa, HaloTag construct with no cDNA: 34 kDa, Full-length NPAS3: 101 kDa. **c**, **d** Luciferase reporter expression demonstrating transactivation function of all three variants tested. The previously characterized variant p.Val304Ile was tested in two isoforms in (**c**). The remaining variants, p.Ala552Pro and p.Gly697Ser was tested in isoform 1. Reporter used is luciferase (*luc2*) expression driven by a construct including 797 bp proximal to the transcription start site of *VGF*. RLU = relative luciferase units, the ratio of firefly luciferase and renilla luciferase to normalize for transfection efficiency. Top and bottom of each box indicate the 25th and 75th centile, internal bar indicates the median, whiskers indicate data extremes. Data are representative of three biological replicates. *p < 0.05, **p < 0.01, ***p < 0.001

Multiple coding variants of NPAS3 have been identified in human studies, including rare and common variants associated with psychiatric illness (p.Val304Ile [12] and p.Ala552Pro [13], respectively). We functionally assessed these variants using the developed assays, as well as a rare population variant, p.Gly697Ser [13]. The rare psychiatric illness-associated p.Val304Ile is localized between the PAS domains of NPAS3 and has been shown to be associated with aggregation, further, it can be hypothesized to affect protein interactivity and function (Fig. 1a, [40]). Although our assays were not designed to assess protein aggregation, we did not observe alterations in expression, localization, deficits in interactivity with ARNT or transactivation function due to this variant in our assays (Figs. 8, 9a–c). For the schizophrenia-associated variant p.Ala552Pro, localized to the transactivation domain, we did not observe functional consequences in any of our assays (Figs. 8, 9a, b, d). For the rare variant localized to a poly-glycine repeat in the transactivation domain not associated with disorder, p.Gly697Ser, we observed normal expression, localization and transactivation function when expressed in isolation, however, co-expression with ARNT resulted in reduced transactivation output (Figs. 8, 9a, b, d).

## Discussion

Through these studies we have experimentally demonstrated that NPAS3 acts as a true transcription factor, behaving as predicted for a bHLH–PAS family member. NPAS3 has been characterized as a bHLH–PAS transcription factor requiring ARNT as an obligate heterodimeric partner, however, the interaction between NPAS3 and ARNT had not been experimentally demonstrated until recent studies of murine Npas3 and Arnt [22, 23]. Our data confirm that the interaction is conserved for human NPAS3 and ARNT, and that it requires both the bHLH and PAS domains. We observe similar effects of ARNT expression on NPAS3 expression as observed for NPAS1, except NPAS3 is much more strongly associated with the nucleus than NPAS1 [49]. As NPAS3 has been observed to be variably localized to the nucleus by immunohistochemistry and immunofluorescence, this may indicate that subcellular localization of NPAS3 is an active process that is regulated in response to environmental or cellular stimuli [12, 14, 25, 50]. Interestingly, we have observed an effect of ARNT expression on the localization of the NPAS3 PAS domain construct in isolation, which we did not observe to be able to interact with ARNT in our pull-down studies. This may be due to the detection of a weak residual interactivity in *in cellulo*, which is not of high enough affinity to be captured by our pull down assay. Alternately, expression of ARNT may enhance nuclear localization of the PAS domain of NPAS3 by interacting with and saturating the pool of a cytoplasmic protein involved in the regulation of bHLH–PAS protein localization and preventing it from restricting the NLS-deficient PAS construct to the cytoplasm.

We have experimentally validated the categorization of NPAS3 as a transcription factor through assessment of its ability to bind DNA and cause regulation of target genes. To this end, we co-registered ARNT ChIP-seq data with previously generated microarray data screening for genes differentially regulated by NPAS3 [25, 30]. Of the 14 genes identified as potential co-targets, two were found to be regulated by expression of NAPS3 and ARNT: *TXNIP* and *VGF*. The regulation of *VGF* as a target of NPAS3 has been assessed previously, however, the contribution of ARNT to this regulation has not been studied [25, 26, 51]. Similar to other groups, we observe that expression of NPAS3 results in activation of reporter expression driven by *VGF* promoter constructs. Our construct is limited to the 797 bp of the proximal *VGF* promoter, excluding most of exon 1 and intron 1 entirely, and expression of NPAS3 results in the same magnitude of activation as the larger constructs used by other groups. Combined with our ChIP data, we have shown that NPAS3 associates with the proximal promoter region to regulate *VGF* expression. We further find that co-expression of NPAS3 and ARNT results in additive effects on *VGF* promoter driven expression.

Repression of *TXNIP* by NPAS3 was replicated in our studies but only with co-expression of NPAS3 and ARNT [25]. In other experiments, we have observed that *TXNIP* mRNA expression increases over time in culture (data not shown). As TXNIP is rapidly regulated to maintain metabolic homeostasis and apoptotic response to multiple environmental stresses and cues, this may represent a cellular response to altered metabolic state and increased propensity to apoptosis in the face of environmental stress due to media depletion, pH changes, reduced levels of nutrients, increases in metabolic by-products and oxidative stress over time in culture [52–56]. NPAS3 may inhibit the induction of *TXNIP* in order to promote cellular survival, similar to its pro-neurogenic role in the hippocampus [15]. NPAS3 was found to associate with a region proximal to the promoter of *TXNIP*. *TXNIP* regulation was found to require co-expression of NPAS3 and ARNT, however, the region including the 867 bp 5′ of the transcription start site was found not to be sufficient for repression of reporter expression. Exploration of other binding sites proximal to this region, including exon 1/intron 1 should be undertaken in order to further characterize the nature of the repressive complex. ARNT may bind at a distal site to facilitate repression, which may explain why we observe binding of NPAS3 to the *TXNIP* promoter in the absence of expressed ARNT and gene regulatory output. Although our construct contains variants relative to the genomic locus, these variants are outside of predicted ARNT binding sites, and the construct was found to be expressive, and normally responsive to glucose, suggesting that these variants do not significantly affect function [57, 58].

A recent ChIP-seq study of the mouse hippocampus, where deletion of *Npas3* has been associated with loss of neurogenesis, did not identify *VGF* or *TXNIP* as differentially regulated by loss of NPAS3, nor bound by NPAS3 in wild-type mouse hippocampus [27]. This may represent a difference due to differences in methodology and systems assessed, as we have used an in vitro human cell system. As this model system is markedly different in cell type (monoculture of human cells derived from embryonic kidney) and cultured in synthetic medium, the gene regulatory responses elicited by NPAS3 expression may vary markedly, potentially due to environmental factors as well as varying expression of potential gene regulatory co-factors that affect gene regulation by NPAS3. We selected HEK 293T cells as they are directly related to the cells the original study used to identify NPAS3-regulated genes in order to assess the mechanism of regulation by NPAS3 that results in the observed changes in gene expression [25]. Transcriptomic profiling suggests that these cells are of neural crest origin and express neural genes [59]. Further, *Npas3* is expressed in non-neural cells and its gene-regulatory mechanism is relevant to multiple tissue- and cell-types [2, 24, 60]. The non-replication may also be contributed to by variable regulation in response to environmental stimuli, as we observe regulation of *TXNIP* only under stress conditions. *TXNIP* has been shown to be rapidly and transiently regulated in response to cellular stimuli, as such occupancy of NPAS3 at this locus may not be constitutive [61]. As TXNIP is involved in cellular redox balance, and response of cells to intra- and extracellular stressors, affecting inflammatory tone, metabolism and apoptotic pathways, NPAS3 may contribute to cellular survival by repressing the pro-apoptotic function of TXNIP [54, 55, 62, 63].

Using the assays developed to characterize full-length NPAS3 and its interacting partner, ARNT, we have characterized the predicted functional domains of NPAS3. The bHLH domain was found to be critical for interaction with ARNT, as well as for specificity of the regulatory action of NPAS3. The bHLH domain contains a predicted nuclear export sequence and appears to contribute to the subcellular localization of NPAS3. Both the PAS and the bHLH domains were found to be critical for heterodimerization with ARNT, consistent with the observed interaction interfaces identified in the crystal structure of Npas3::Arnt and other bHLH–PAS heterodimers [23, 64, 65]. The PAS(A) domain is considered to be critical for the specific and high-affinity interaction of bHLH–PAS proteins with ARNT [20, 21] while the PAS(B) domains of bHLH–PAS proteins are critical for normal gene regulatory function, ligand binding and protein::protein interactions with chaperone proteins [21, 66–69]. Both PAS(A) and PAS(B) domains have been shown to be involved in interactions with co-activators of bHLH–PAS proteins [70–72]. Although we did not assess the PAS(A) and PAS(B) repeats independently, other groups have assessed a construct encoding the bHLH and PAS(A) domain independently and find that it can effect muted gene regulatory function relative to full-length NPAS3 [25, 26, 51]. The reduced gene regulatory output is likely contributed to by loss of coactivators potentially recruited by the PAS(B) and C-terminal transactivation domain, and potentially due to reduced interactivity with heterodimeric partners, such as ARNT. Our data demonstrate that the region C-terminal to the PAS(B) domain predominantly contributes to the transactivation function of NPAS3. The bHLH–PAS domain construct studied here acts to repress activation by ARNT, and is unable to cause activation of reporter expression when expressed in isolation. Furthermore, expression of the C-terminus in isolation is sufficient to non-specifically activate reporter gene expression, demonstrating potent transactivation function. These data confirm for the first time that the C-terminus of NPAS3 encodes a true transactivation domain. Through these studies we have confirmed the function of the domains of NPAS3, and have experimentally demonstrated that NPAS3 acts as a true transcription factor.

Finally, we assayed three variants identified in the human population for effects on NPAS3 function. The psychiatric disorder-associated variants p.Val304Ile and p.Ala552Pro were found to be normally expressed and localized to the nucleus, and further, did not affect interaction with ARNT, nor regulation of the *VGF* reporter construct relative to wild-type. Previous studies have identified that the p.Val304Ile variant, which has been found to be sequestered in the insoluble fraction of cell lysates, suggestive of aggregation [51]. This variant has been observed in various populations at low frequency [Exome Aggregation Consortium (ExAC) worldwide minor allele frequency (MAF) = 0.0001] [73]. Our studies were not designed to assess aggregation, however, we found no reduction in its ability to activate expression driven the *VGF* promoter which is in conflict with previously observed deficits [51]. As such further study of this variant is warranted to validate the effects of this variant on NPAS3 function.

Since its association with schizophrenia, the p.Ala552Pro variant has since been found at similar frequencies in unselected and normal control populations (ExAC worldwide MAF = 0.14) [13, 17, 73]. We undertook studies to functionally characterize this variant due to the nature of the amino acid substitution, a large proline residue in place of an alanine, which have been shown to be poorly tolerated [74]. We did not observe any functional effect of this variant, which may be expected due to its presence in the normal population. Our assays may not be sensitive enough to detect the functional significance of this variant, or may not be designed in such a way as to detect the effects, for example, the predicted alteration to splicing enhancers [13].

Finally we assessed the low frequency variant p.Gly697Ser, which has not been associated with disorder, but is present in the normal population at a low frequency (ExAC worldwide MAF = 0.0013) [13, 73]. This variant was found to be normally expressed and localized to the nucleus. Expressed individually, the transactivation function of this variant was found to be normal, however, when co-expressed with ARNT, it was found not to cooperatively activate expression driven from the *VGF* promoter. This variant is localized to a poly glycine repeat within the transactivation domain which has been expanded in humans [13, 18]. Although the function of poly-glycine repeats is poorly characterized, it is thought to be involved in spacing of functional domains and may contribute to protein::protein interactions, based on observations of aggregation associated with large expansions [75–77]. Variants as small as 1 amino acid deletions in poly-glycine repeats have been shown to affect protein function, and have been associated with human disorders [78–80]. As such this variant may contribute to variation in NPAS3 transactivation function, potentially by affecting interaction with a co-activator of the NPAS3::ARNT heterodimer, or other functional NPAS3 complex.

## Conclusions

Through these studies we have demonstrated that NPAS3 acts as a transcription factor. Furthermore, we have experimentally validated the function of the bHLH domain in DNA binding, the PAS domains in interaction with ARNT, and C-terminal tail as a potent transactivation domain. In order to expand our understanding of variation to NPAS3, we characterized the functional significance of variants identified in the human population. We found that both the previously psychiatric disorder-associated variants, p.Val304Ile and p.Ala552Pro variants did not have significantly altered molecular function. However, we identified altered transactivation function for the rare population variant p.Gly697Ser when co-expressed with ARNT. These data expand our understanding of the molecular function of NPAS3, as well as the contribution of variants to NPAS3 in its gene regulatory function and are important for interpretation of variants identified in next generation sequencing studies of individuals.

## Additional files

**Additional file 1: Figure S1.** Validation of NPAS3 antibody. (A) Western blot demonstrating detection of both NPAS3 constructs used in this study, with co-detection by the HA antibody. (B) Western blot demonstrating detection of expressed NPAS3, as well as a 100 kDa band in SK-N-SH cells known to express NPAS3, no NPAS3 antibody bands are co-detected by the NPAS1 antibody. (C) Immunoprecpititation data demonstrating that the NPAS3 antibody can immunoprecipitate expressed NPAS3 constructs. (D) Immunofluorescence with the NPAS3 antibody detected predominantly nuclear signal which did not overlap with NPAS1 signal, which was predominantly cytoplasmic. 1000X magnification, scale bar = 5 μm.

**Additional file 2: Table S1.** Primers used for qPCR analysis of gene expression in this study. **Table S2.** Primers used for ChIP PCR analysis.

**Additional file 3: Figure S2.** Immunofluorescence images of NPAS3 domains. Immunofluorescence microscopy images of cells expressing NPAS3 domain constructs in the presence and absence of co-expressed ARNT. HEK 293T cells were transfected with indicated constructs and incubated for 48 hours prior to fixing with paraformaldehyde and probing for ARNT and HA (NPAS3 domain constructs), and stained with DAPI to indicate nuclei. 400X magnification, scale bar = 10 μm.
