# Supplementary figures and images for: Molecular analysis of NPAS3 functional domains and variants
Source: BMC Mol Biol. 2018 Dec 3;19:14. doi: 10.1186/s12867-018-0117-4 (PMC6276216; doi:10.1186/s12867-018-0117-4)

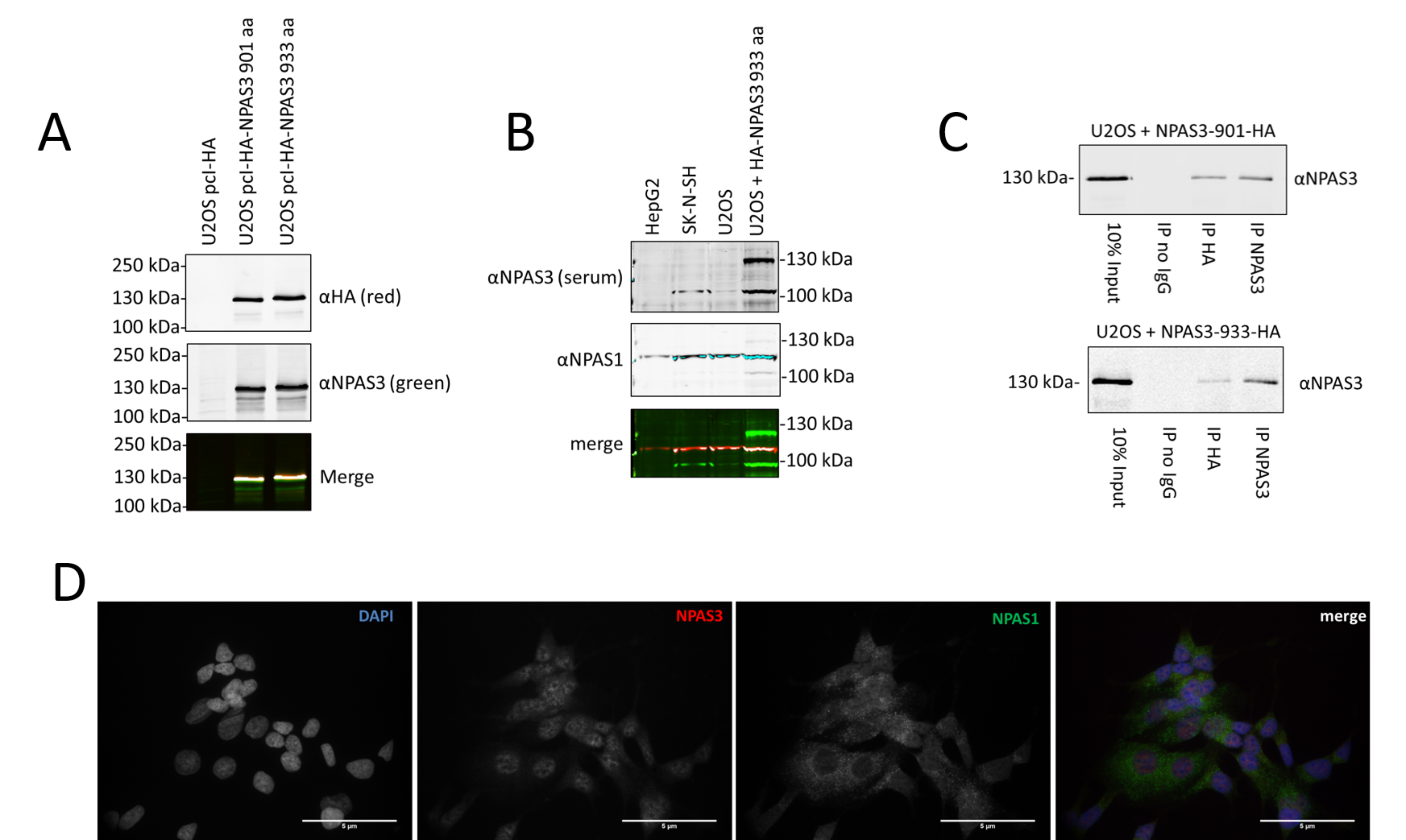

Supplement: Supplementary file 1 — Additional file 1: Figure S1. Validation of NPAS3 antibody. (A) Western blot demonstrating detection of both NPAS3 constructs used in this study, with co-detection by the HA antibody. (B) Western blot demonstrating detection of expressed NPAS3, as well as a 100 kDa band in SK-N-SH cells known to express NPAS3, no NPAS3 antibody bands are co-detected by the NPAS1 antibody. (C) Immunoprecpititation data demonstrating that the NPAS3 antibody can immunoprecipitate expressed NPAS3 constructs. (D) Immunofluorescence with the NPAS3 antibody detected predominantly nuclear signal which did not overlap with NPAS1 signal, which was predominantly cytoplasmic. 1000X magnification, scale bar = 5 μm. [file 12867_2018_117_MOESM1_ESM.tif]

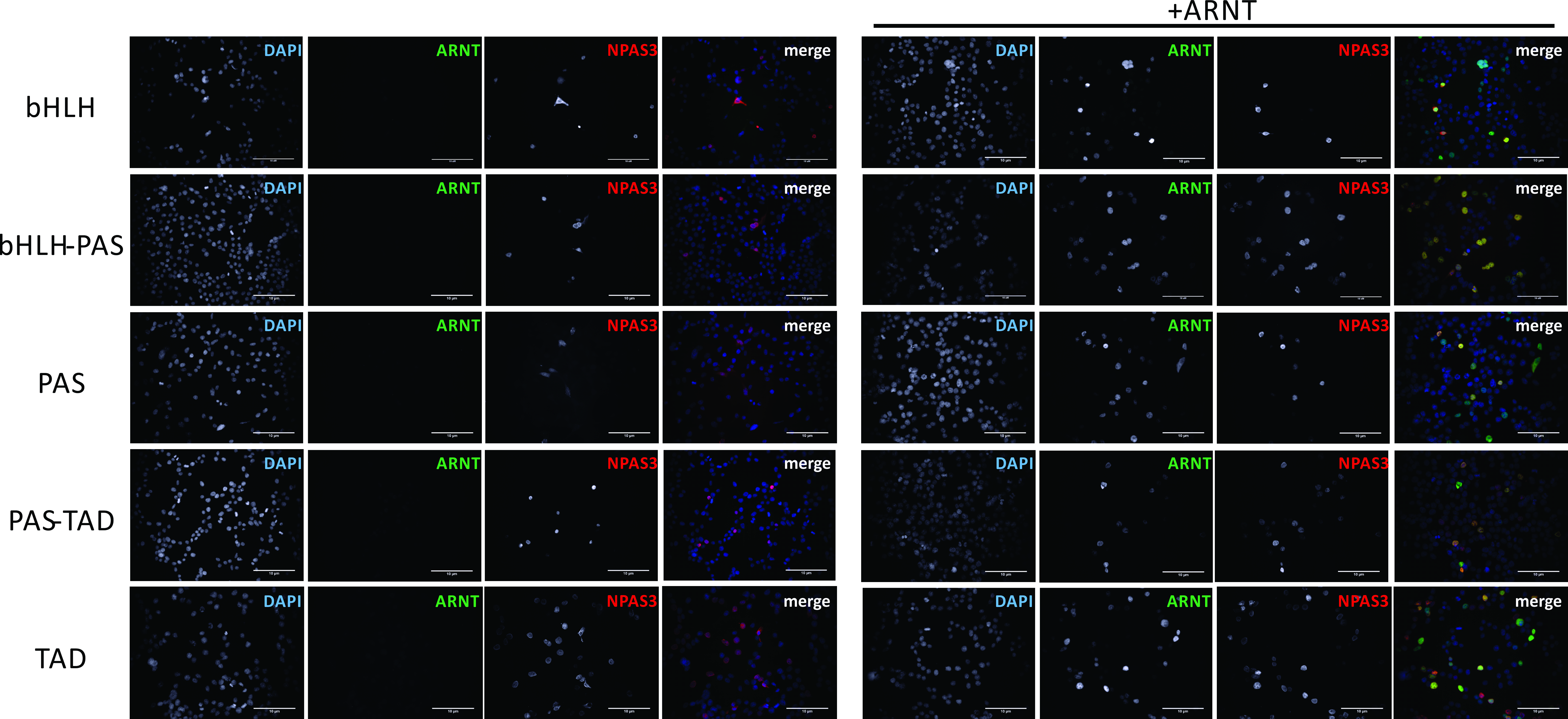

Supplement: Supplementary file 3 — Additional file 3: Figure S2. Immunofluorescence images of NPAS3 domains. Immunofluorescence microscopy images of cells expressing NPAS3 domain constructs in the presence and absence of co-expressed ARNT. HEK 293T cells were transfected with indicated constructs and incubated for 48 hours prior to fixing with paraformaldehyde and probing for ARNT and HA (NPAS3 domain constructs), and stained with DAPI to indicate nuclei. 400X magnification, scale bar = 10 μm. [file 12867_2018_117_MOESM3_ESM.tif]
