# Supplementary material for: Molecular analysis of NPAS3 functional domains and variants
Source: BMC Mol Biol. 2018 Dec 3;19:14. doi: 10.1186/s12867-018-0117-4 (PMC6276216; doi:10.1186/s12867-018-0117-4)
Supplement: Supplementary file 2 — Additional file 2: Table S1. Primers used for qPCR analysis of gene expression in this study. Table S2. Primers used for ChIP PCR analysis. [file 12867_2018_117_MOESM2_ESM.docx]

**Additional Tables**

| Table S1: Primers used for qPCR analysis of gene expression in this study   \| Primer name \| Sequence \| \| --- \| --- \| \| HMBS-qPCR-F ^a^ \| GGCAATGCGGCTGCAA \| \| HMBS-qPCR-R ^a^ \| GGGTACCCACGCGAATCAC \| \| HPRT1-qPCR-F ^a^ \| TGACACTGGCAAAACAATGCA \| \| HPRT1-qPCR-R ^a^ \| GGTCCTTTTCACCAGCAAGCT \| \| SDHA-qPCR-F ^a^ \| TGGGAACAAGAGGGCATCTG \| \| SDHA-qPCR-R ^a^ \| CCACCACTGCATCAAATTCATG \| \| ANG-qRTPCR-F \| GGATAACTCCAGGTACACACAC \| \| ANG-qRTPCR-R \| CCGTCTCCTCATGATGCTTT \| \| ANKRD37-qRT-PCR-F \| TAGGAGAAGCTCCACTACACA \| \| ANKRD37-qRT-PCR-R \| GCTGTTTGCCCGTTCTTATTAC \| \| ATF5-qRTPCR-F \| GTCTATGCCCGTCACATAACA \| \| ATF5-qRTPCR-R \| CCAGACAACCACCTGTAAGAA \| \| DHCR24-qRTPCR-F \| ACAGCATCAGGTGGGAAAG \| \| DHCR24-qRTPCR-R \| GGGATGAGTGGTTGGAGAAAT \| \| HIST1H4H-qRTPCR-F \| CAAGCGAATTTCTGGCCTTATC \| \| HIST1H4H-qRTPCR-R \| TTTGGCGTGCTCTGTGTAA \| \| MAT2A-qRTPCR-F \| TATCACCCAACGCTCCAAAG \| \| MAT2A-qRTPCR-R \| CATTGCCAGACAGAGGCTATAA \| \| NCLN-qRTPCR-F \| GCCTCAGCTTCCTCATCAATAG \| \| NCLN-qRTPCR-R \| ACCTCCCTCTCTGAGTTCCA \| \| RNASE4-qRTPCR-F \| CTCTGTCTCCTCAGCTCATTTC \| \| RNASE4-qRTPCR-R \| AGCCCAGCCTCATTCATTAC \| \| RPL37-qRTPCR-F \| GGAGTACCACTGGAAACGTATG \| \| RPL37-qRTPCR-R \| CACTTAGCTAGCCACCTTACAC \| \| TXNIPex1-qRTPCR-F \| GTGATAGTGGAGGTGTGTGAAG \| \| TXNIPex1-qRTPCR-R \| CAGGTACTCCGAAGTCTGTTTG \| \| TXNIP-qRTPCR-F \| CGATAGTTTCGGGTCAGGTAAA \| \| TXNIP-qRTPCR-R \| TTGGCTCTTCTCCACATGATAC \| \| USP49-qRTPCR-F \| GACCTTTGCCTATGATCTCTCC \| \| USP49-qRTPCR-R \| CTCCCTCTGTGTTGTAGCAATAG \| \| VGF-qRTPCR-F \| GACCTGCTGCTCCAGTATTT \| \| VGF-qRTPCR-R \| TCCTCCCTTGCACTCTCT \| \| ZBTB40-qRTPCR-F \| TGGAGTTTCTGCTGGAAGTG \| \| ZBTB40-qRTPCR-R \| CCAGGCTTCAGGTAAGGAATAC \| \| ZNF581-qRTPCR-F \| GAATCTGCGCCATCTTCCT \| \| ZNF581-qRTPCR-R \| CAGAGTGGGAAACGTGTTTATTG \|   ^a^ Previously characterized in [1] |
| --- | --- | --- | --- | --- | --- | --- | --- | --- | --- | --- | --- | --- | --- | --- | --- | --- | --- | --- | --- | --- | --- | --- | --- | --- | --- | --- | --- | --- | --- | --- | --- | --- | --- | --- | --- | --- | --- | --- | --- | --- | --- | --- | --- | --- | --- | --- | --- | --- | --- | --- | --- | --- | --- | --- | --- | --- | --- | --- | --- | --- | --- | --- | --- | --- | --- | --- | --- | --- | --- | --- | --- | --- | --- | --- |

| Table S2: Primers used for ChIP PCR analysis   \| Primer name \| Sequence \| Product size \| \| --- \| --- \| --- \| \| TXNIPprom-qPCR-R \| AGGATCCCACTGACCCTAAA \| 139bp \| \| TXNIPprom-qPCR-F \| CTGGCTAAGACTAGGCATGAAA \| \| \| TXNIPex1-qPCR-F \| GTGATAGTGGAGGTGTGTGAAG \| 120bp \| \| TXNIPex1-qPCR-R \| CAGGTACTCCGAAGTCTGTTTG \| \| \| TXNIP-distCDS-F \| GAGTGTGGGTCCACCTTAGC \| 115bp \| \| TXNIP-distCDS-R \| TGTATCACAACATGGGCGCT \| \| \| VGFprom-qPCR-F \| GCGTTGCTGAGTGGAATAGA \| 105bp \| \| VGFprom-qPCR-R \| CTGGTCGGCTCTTGAATCTTTA \| \| \| VGF-intron1-F \| GTGTTTGCAACACCCCCATC \| 92bp \| \| VGF-intron1-R \| AATACTCCGCTGTTCGTCCC \| \| \| VGF-distCDS-F \| TCAGTCCAAGTAGCGCCAAG \| 93bp \| \| VGF-distCDS-R \| GGCTCTCCAGATTCACTCGG \| \| |
| --- | --- | --- | --- | --- | --- | --- | --- | --- | --- | --- | --- | --- | --- | --- | --- | --- | --- | --- | --- | --- | --- | --- | --- | --- | --- | --- | --- | --- | --- | --- | --- | --- | --- | --- | --- | --- | --- | --- | --- |

Additional References

1.Vandesompele J, De Preter K, Pattyn F, Poppe B, Van Roy N, De Paepe A, Speleman F: **Accurate normalization of real-time quantitative RT-PCR data by geometric averaging of multiple internal control genes.** Genome Biol 2002, **3**(7):RESEARCH0034.
